# An Assessment of the Glyconutrient Ambrotose™ on Immunity, Gut Health, and Safety in Men and Women: A Placebo-Controlled, Double-Blind, Randomized Clinical Trial

**DOI:** 10.3390/nu12061751

**Published:** 2020-06-11

**Authors:** Richard J. Bloomer, Matthew Butawan, Marie van der Merwe, Faith H. Keating

**Affiliations:** Center for Nutraceuticals and Dietary Supplement Research, School of Health Studies, 106 Roane Fieldhouse, University of Memphis, Memphis, TN 38152, USA; mbutawan@cbu.edu (M.B.); mvndrmrw@memphis.edu (M.v.d.M.); fhkating@memphis.edu (F.H.K.)

**Keywords:** glyconutrition, immune function, gut health, quality of life, dietary supplements

## Abstract

Background: Certain dietary fibers have been reported to improve gut health and cellular immunity. Ambrotose is a glyconutrient supplement that contains mannose-rich polysaccharides (acemannan), reported to improve immune function. A more nutrient-dense version of this dietary supplement has been developed recently, with added aloe leaf gel powder (acemannan). The purpose of this study was to evaluate the impact of the traditional and newly developed Ambrotose products on immunity, gut health, and psychological well-being in healthy men and women. Methods: Seventy-five men and women were randomly assigned in double-blind manner to one of five treatments, as follows: Ambrotose Advanced (AA) at 2 or 4 g daily, Ambrotose LIFE (AL) at 2 or 4 g daily, or placebo. Subjects ingested their assigned treatment daily for eight weeks. Resting heart rate, blood pressure, and measures of psychological well-being were analyzed before and after four and eight weeks of supplementation. Blood samples were collected at the same times and analyzed for zonulin, hematology measures, and cytokines—IL-6, IL-10, IL-1β, and TNF-α (analyzed both with and without stimulation via lipopolysaccharide [LPS]). Results: All Ambrotose treatments were well-tolerated. There were no differences among treatments in heart rate or blood pressure across time. Self-reported well-being scores were generally higher for the Ambrotose treatments but there were no changes of statistical significance across time (*p* > 0.05). Differences of statistical significance were noted for select biochemical variables, the most notable being a dramatic decrease in monocytes in the Ambrotose groups. No change was noted in the cytokine response to LPS stimulation in all groups, indicating a maintenance of a healthy immune response. Conclusion:Regular supplementation with Ambrotose is safe and can improve subclinical cellular adversity (as evidenced by a decrease in monocytes), without unnecessary activation of an immune response.

## 1. Introduction

Increasing evidence supports the role of glyconutrients such as dietary fibers in modulating the gut microbiome, thereby regulating multiple physiological functions ranging from immunity to cognitive processes [1]. The product known as Ambrotose is a blend of glyconutrients that has been used by human subjects for several years, with noted benefits of enhanced immunity [2], improved cognitive performance [3,4,5], and enhanced antioxidant capacity [2,6]. These outcomes may be indirectly impacted by alterations to the gut microbiota (microbiome) through intake of dietary fiber [7].

In vitro studies suggest that Ambrotose is a promising prebiotic, which may positively promote gut health [8,9]. Anecdotal reports of improved mood and well-being are common with Ambrotose supplementation. Recent evidence suggests that dietary fiber-induced microbiome changes may impact these subjective measures [10].

Previous studies indicate that daily ingestion of Ambrotose enhances antioxidant capacity at rest and following exercise [2,6], increases phagocytic activity of granulocytes [2], and increases serum protein N-glycosylation [11,12]. Additionally, acute supplementation with Ambrotose has resulted in improvements in recognition and working memory [4]. In the work to date, involving approximately 350 participants and lasting up to six months, the treatment with Ambrotose has been very well tolerated, with adverse events limited to “mild” or “self-limiting” or absent altogether [2,4,6,11,12].

The typical daily dosage of Ambrotose consumed by individuals both within and outside of a research setting ranges from 2–4 g, with dosing established based on in-house evaluations with acemannan, coupled with anecdotal findings of reported “improved health” and data specific to customer use statistics. No study to date has evaluated whether or not one dosage is more efficacious than another. Moreover, while Ambrotose has been used successfully for the past several years, a new formula of the product has been developed and is believed to offer a greater benefit to consumers, due to the increase quantity of aloe leaf gel powder, as well as other ingredients noted in the Methods section. In the present study, we compared the efficacy of daily Ambrotose ingestion at either 2 or 4 g daily, using either the traditional “Advanced” Ambrotose or a new Ambrotose “LIFE” version of the product with regards to bloodborne variables of immunity and gut health, as well as subjective well-being measures.

## 2. Methods

### 2.1. Subjects

A total of 75 men and women participated in this study. Subjects were required to: be aged 20–65; have no diagnosis of cardiovascular, metabolic, or neurological disease; not be using nutritional supplements or medications known to impact immunity or gut health; be physically active by participating in structured exercise at least twice per week for 30 or more minutes per session; not be pregnant. Health history, medication and dietary supplement usage, and physical activity questionnaires were completed by all subjects and baseline anthropometric measures were obtained. Female subjects were required to take a urine pregnancy test to confirm that they were not pregnant. Subject characteristics are presented in Table 1. Prior to participation, each subject was informed of all procedures, potential risks, and benefits associated with the study through verbal and/or written form in accordance with the procedures approved by the University Institutional Review Board for Human Subjects Research (#PRO-FY2018-488). Subjects provided written informed consent prior to being admitted into the study.

### 2.2. Treatment Assignment

Subjects were randomly assigned into one of five treatment groups with equal numbers per group (*n* = 15). Specifically, subjects were assigned to ingest either 2 or 4 g per day of Advanced Ambrotose (AA), Ambrotose LIFE (AL), or a placebo. Advanced Ambrotose contains the following ingredients: aloe vera extract inner leaf gel (containing acemannan), arabinogalactan, ghatti gum, glucosamine HCL, gum tragacanth, vitamin A, beta carotene, wakame algae extract, and rice starch. Ambrotose LIFE contains similar ingredients as noted above, with a higher amount of aloe vera extract inner leaf gel and the additional ingredients, RiFiber (rice bran) and Modified Citrus Pectin with Sodium Alginate. We believed that the additional acemannan would promote a beneficial effect on the immune response, as indicated by some of our blood borne measures. Note: the acemannan used in this study complied with the current definition of acemannan as required by the Chemical Abstract Service #110042-95-0 and the American Medical Association’s United States Adopted Names Council (USAN). The placebo was maltodextrin. It should be noted that there was no positive control used in this design (such as acemannan alone) and this may be viewed as a limitation. All treatments were provided in powder form in unlabeled bottles and subjects were instructed to ingest either one scoop (2 g dose) or two scoops (4 g dose) daily for eight weeks. Subjects were instructed to mix the powder in a beverage and consume. The powder was weighed before providing to the subjects and upon bottle return, in order to calculate compliance to intake. Subjects were required to wash-out from any current nutritional supplements that may have impacted outcome measures, for a period of four weeks prior to the start of the study. Women began the study during the first five days of their menstrual cycle, in an attempt to control for circulating hormones.

### 2.3. Laboratory Test Visits

Subjects reported to the lab in the morning hours following an overnight fast (no food or calorie containing beverages after 10 pm). They reported on three separate occasions, over an eight-week period: baseline, after four weeks, and after eight weeks of supplementation. Each test visit lasted approximately 45 min. At each visit, subjects arrived at the lab, rested quietly for 10 min and then had a blood sample taken. Subjects also turned in their diet logs, completed during the five days prior to each test visit (described below). Subjects completed three quality of life/subjective feeling questionnaires, as indicated below.

### 2.4. Health and Well-Being Questionnaires

The self-reported assessment of general well-being (SF-12) is a commonly used 12-item questionnaire used to measure functional health and well-being from the subject’s point of view [13]. The self-reported Psychological General Well-Being Index (PGWBI) is another widely used scale to assess well-being from a subject’s perspective [14]. The self-reported assessment of fatigue and associated variables is a visual analog scale in which the subject is asked to make a mark on a 100-mm scale indicating how he/she feels in regards to the variable in question. These questionnaires were used to assess if any changes in subjective mood or well-being occur with supplementation.

### 2.5. Blood Collection and Analysis

Approximately 15 mL of blood was taken from subjects at the times indicated above after an overnight fast. Samples were collected into BD Vacutainer™ tubes containing lithium heparin (BD, Catalog # BD367880) and BD Vacutainer Plus™ for serum collection. White blood cell numbers were determined at time of blood collection using a Hematology analyzer (VetScanHM2, Abaxis). Whole blood was centrifuged, and serum/plasma immediately removed and stored at −80 °C. Serum Zonulin concentrations were quantitated using a commercially available kit (ALPCO, Catalog # 30-ZONSHU-E01) and following the manufacturer’s instructions.

### 2.6. Ex Vivo Stimulation LPS Stimulation and Cytokine Measurements

For ex vivo immune stimulation, blood collected in heparinized tubes was stimulated with the Toll-like receptor (TLR) ligand lipopolysaccharide (LPS) within 60 min of blood collection. Briefly, 220 uL of whole blood was incubated in 96-well U bottom plates with 30 uL RPMI or RPMI containing LPS (*Escherichia coli*, Novus Biologicals, Centennial, CO, USA) at a final concentration of 250 ng/mL. Cultures were incubated for 6 h at 37 °C in an environment containing 5% CO_2_. After incubation, supernatants were collected after centrifugation and stored at −80°C until analysis. Cytokine concentrations for Interleukin (IL)-10, IL-6, IL-1β, and TNF-α were determined using the Millipore multiplex magnetic bead system according to the manufacturer’s instruction and a Magpix^®^ analyzer (Luminex Corp, Austin, TX, USA).

### 2.7. Activity and Dietary Intake

Subjects were asked to maintain their usual activity patterns throughout the study period but refrain from strenuous physical activity during the 48 h prior to each test day visit. Dietary intake should have remained similar over the entire study period, but no alcohol should have been consumed during the 48 h prior to each lab test day. Diet records were maintained for five-day periods prior to each test day. Dietary data was analyzed for total calories, macro- and micro-nutrient composition using Food Processor Pro software (Esha Research, Salem, OR, USA).

### 2.8. Data Analysis

Values for all variables were calculated and the data are presented as mean ± SD (as well as mean ± SEM for figures only: for ease of viewing figures). Data were analyzed using a treatment × time analysis of variance, with subsequent Tukey post-hoc testing as needed. Analyses were performed using JMP^®^ Pro software (SAS, Cary, NC, USA) and GraphPad Prism (San Diego, CA, USA) version 8. Significance difference from baseline (Time = 0) was determined by two-way ANOVA for WBC, lymphocyte%, granulocyte%, monocyte%, and monocyte absolute number. For all others, a mixed-effects analysis was used. Statistical significance was determined as *p* < 0.05.

## 3. Results

All 75 participants completed the study and no participant reported an adverse event or problem associated with use of the products, with the exception of one female subject who was assigned to AL2 and reported gastrointestinal discomfort while using the supplement. This subject decided to stop taking the supplement after six weeks, however, she did complete the remainder of the study procedures. Compliance to treatment was as follows: 86 ± 19% for AA4; 88 ± 21% for AA2; 100 ± 27% for Placebo; 78 ± 21% for AL4; 78 ± 14% for AL2. Compliance was higher for Placebo as compared to all other groups (*p* < 0.05) but not different between any of the Ambrotose groups. As the Ambrotose was well-tolerated, we are uncertain as to why compliance to the Placebo was better than that of the other treatments. Of the 225 blood samples (75 subjects × 3 time points), only one blood sample was missing (subject 2 [AA2] at week 8). In addition, as indicated latter in section, some samples when analyzed for the various cytokines were not detectable due to being either too low or too high as compared to the assay range. This was particularly the case for IL-6.

With regard to descriptive variables, no differences of statistical significance were noted between treatments (*p* > 0.05). Data for descriptive variables are reported in Table 1, with associated *p* values included for each variable. Dietary data were not different between treatments or across time (*p* > 0.05). Dietary data are presented in Table 2.

### 3.1. Heart Rate and Blood Pressure

Data for heart rate (HR), systolic (SBP), and diastolic (DBP) blood pressure are presented in Table 3. A treatment effect was noted for HR (*p* = 0.009), with values for placebo higher than for AA2. A treatment effect was also noted for DBP (*p* = 0.002), with values for placebo and AL4 higher than for AA2. For both HR and DBP, baseline values were lowest for AA2, which drove the findings for the treatment effects. No other effects of statistical significance were noted (*p* > 0.05).

### 3.2. Subjective Measures/Quality of Life

The overall PGWBI results for weeks 0 (baseline), 4, and 8 are as follows: 84.7 ± 9.8, 84.1 ± 8.6, 83.7 ± 10.0 for AA4; 85.7 ± 10.8, 88.7 ± 8.5, 90.2 ± 8.7 for AA2; 83.0 ± 9.1, 77.0 ± 15.6, 79.9 ± 14.8 for Placebo; 85.3 ± 11.0, 85.2 ± 12.1, 86.4 ± 11.1 for AL4; 87.4 ± 11.2, 82.6 ± 13.7, 85.5 ± 11.6 for AL2. A treatment effect was noted (*p* = 0.01), with values higher for AA2 than for Placebo. Contrasts revealed that all Ambrotose treatments yielded higher values as compared to Placebo. No differences of significance were noted for the various subcategories of the PGWBI except for the following: a treatment effect was noted for energetic (*p* = 0.03), with higher values noted for AL2 (66.3) as compared to AA4 (52.5). A treatment effect was noted for lethargic (*p* = 0.03), with higher values noted for AA4 (33.0) as compared to AA2 (19.2). A trend for a treatment effect was noted for enthusiastic (*p* = 0.06), with higher values noted for AA4 (58.3), AA2 (59.6), AL4 (63.1), and AL2 (68.0) as compared to Placebo (55.1). A time effect was noted for enthusiastic (*p* = 0.05), with values highest at week 8 (65.8) as compared to weeks 0 (57.9) and 4 (58.7). A time effect was also noted for well-rested (*p* = 0.05), with values highest at week 8 (61.5) as compared to weeks 0 (53.2) and 4 (52.6).

SF-12 results for weeks 0 (baseline), 4, and 8 are as follows: 41.4 ± 2.6, 41.8 ± 2.0, 41.1 ± 2.5 for AA4; 42.3 ± 2.5, 41.3 ± 2.1, 41.9 ± 1.9 for AA2; 42.8 ± 1.6, 42.3 ± 2.1, 41.9 ± 2.6 for Placebo; 42.2 ± 1.8, 41.1 ± 2.9, 41.3 ± 2.4 for AL4; 41.1 ± 2.1, 41.1 ± 3.0, 40.1 ± 2.4 for AL2. A treatment effect was noted for SF12 (*p* = 0.03), with values higher for Placebo than for AL2. No other effects were noted (*p* > 0.05), as values were very similar between groups and across time.

Data for the self-reported assessment of fatigue and associated variables using a visual analog scale are presented in Table 4. No treatment, time, or treatment x time effects were noted for any subjective variable (*p* > 0.05).

### 3.3. Biochemical Variables

**Figure 1:**
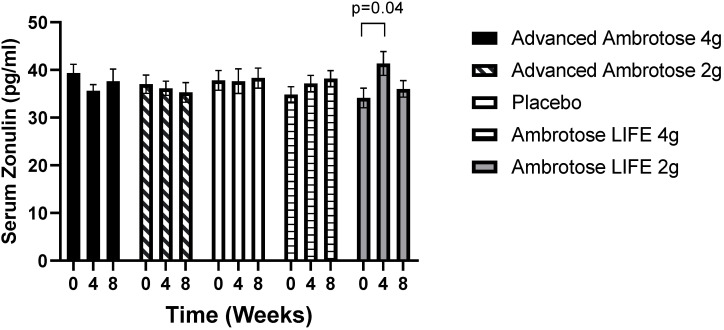
Serum Zonulin concentrations of healthy men and women at baseline and four and eight weeks after supplementation. Values are mean ± SEM, *n* = 15 per group. Ambrotose LIFE 2 g was significantly different between baseline and four weeks of supplementation (*p* = 0.04).

**Figure 2:**
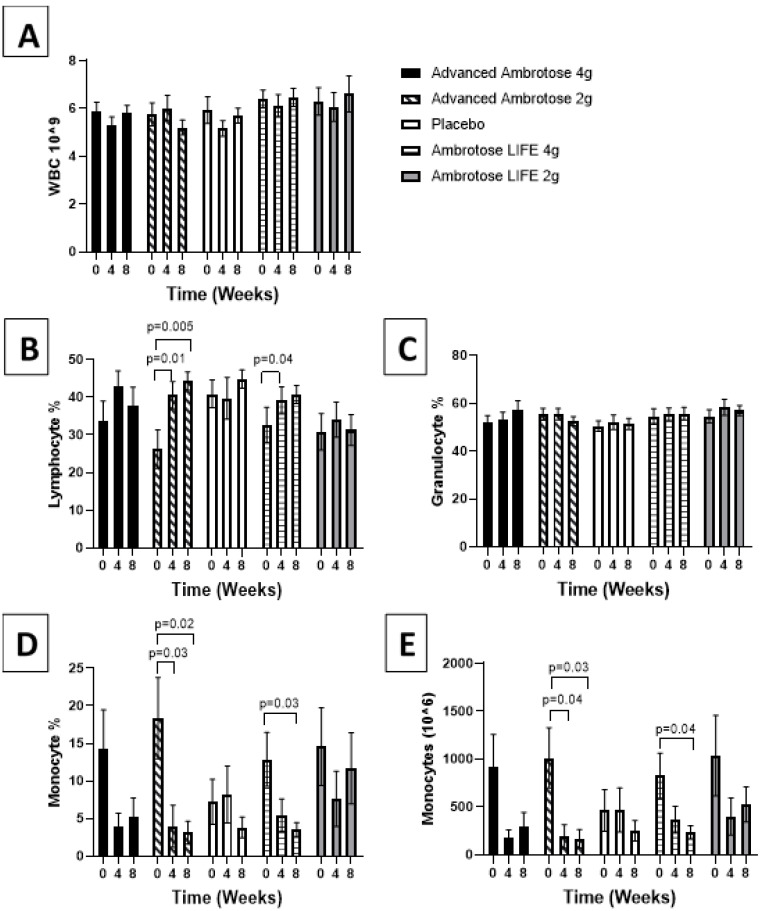
Hematological measurement determined at baseline and at four and eight weeks after supplementation. (**A**) Total white blood cell count. Fraction of lymphocyte (**B**), granulocytes (**C**), monocytes (**D**) of total circulating white blood cells. (**E**) Absolute monocyte count was calculated. Values are mean ± SEM, *n* = 15 per group. Significant changes are indicated where *p* < 0.05.

**Figure 3:**
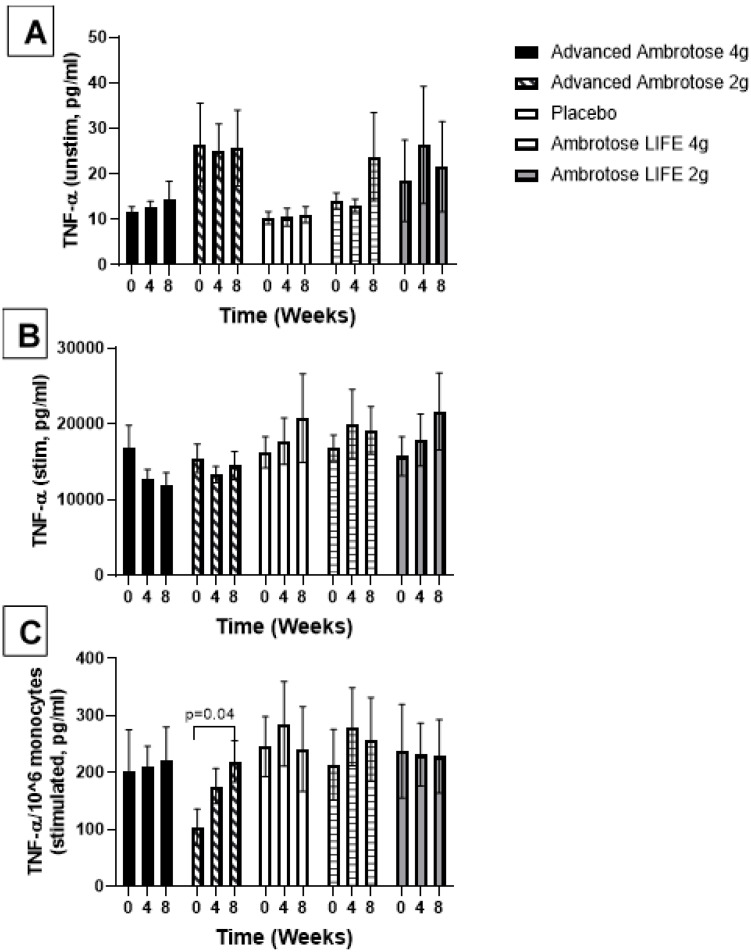
Plasma TNFα without stimulation (**A**) and with lipopolysaccharide stimulation (**B**). (**C**) Stimulated samples are normalized to monocyte numbers. Values are mean ± SEM, *n* = 15 per group. Significant changes are indicated where *p* < 0.05.

**Figure 4:**
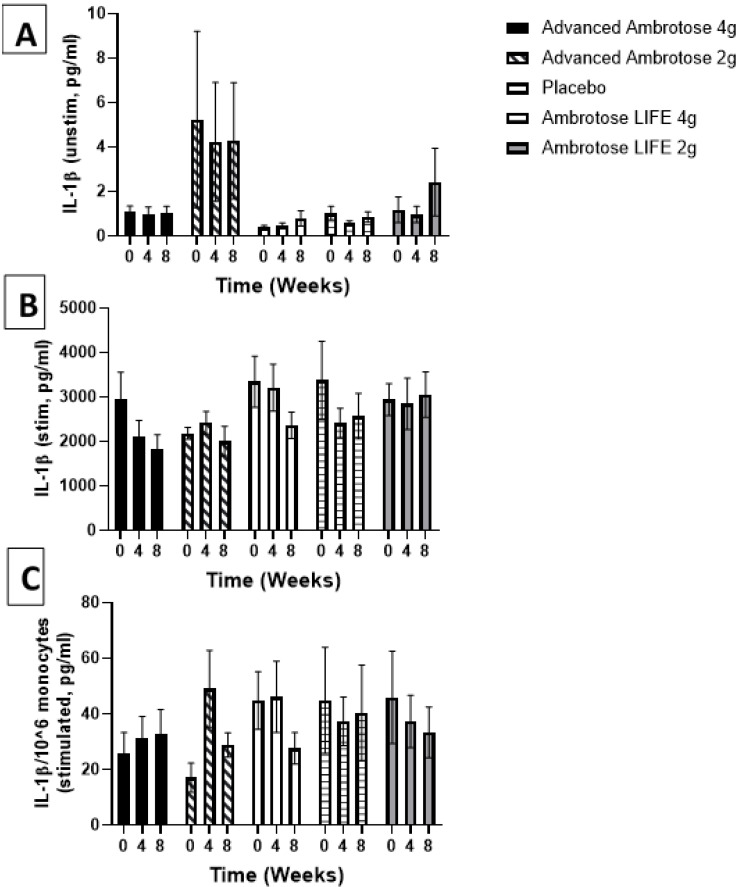
Plasma IL-1β without stimulation (**A**) and with lipopolysaccharide stimulation (**B**). (**C**) Stimulated samples normalized to monocyte numbers. Values are mean ± SEM, *n* = 15 per group. Significant changes are indicated where *p* < 0.05.

**Figure 5:**
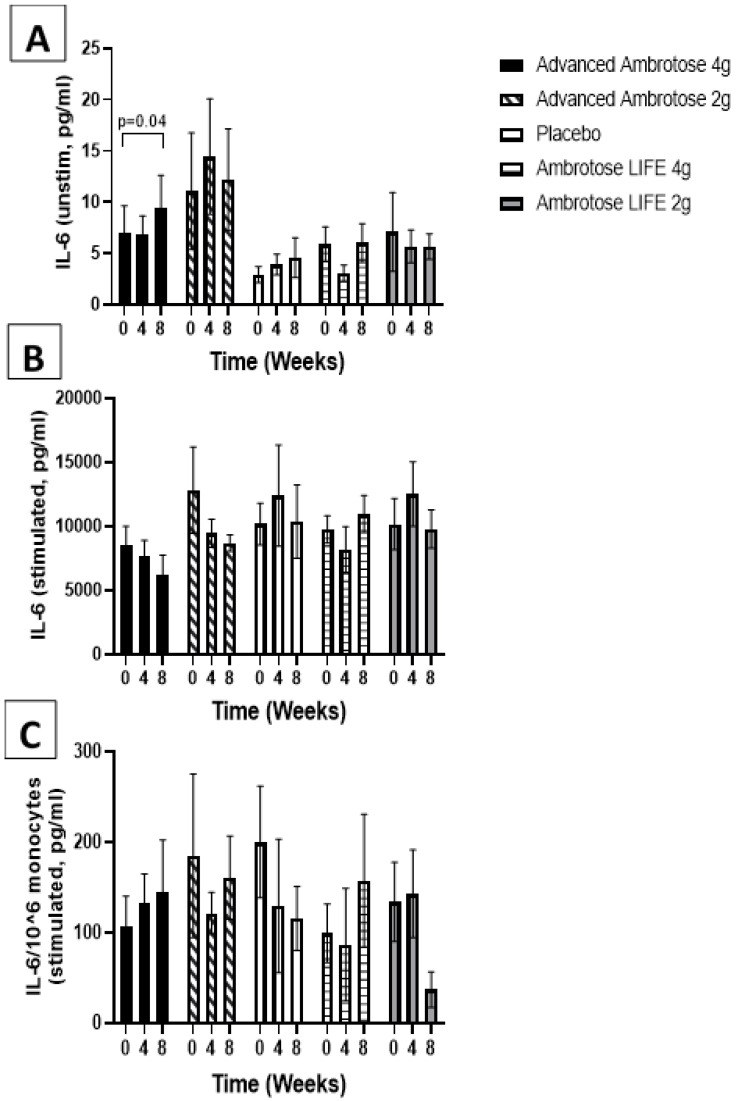
Plasma IL-6 without stimulation (**A**) and with lipopolysaccharide stimulation (**B**). (**C**) Stimulated samples normalized to monocyte numbers. Values are mean ±SEM, *n* = 15 per group. Significant changes are indicated where *p* < 0.05.

**Figure 6:**
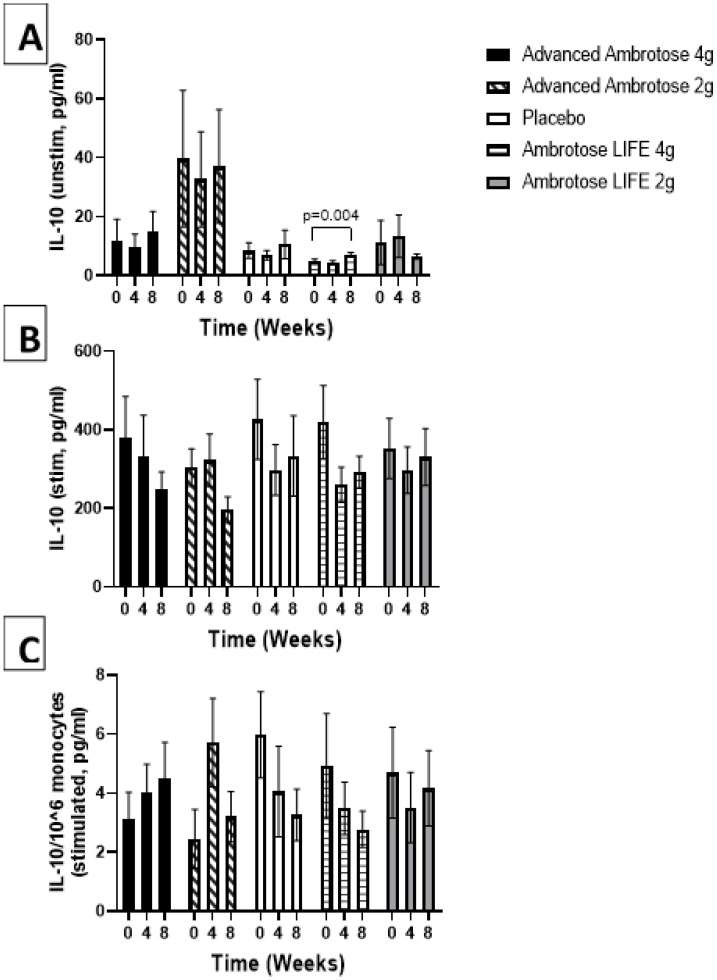
Plasma IL-10 without stimulation (**A**) and with lipopolysaccharide stimulation (**B**). (**C**) Stimulated samples normalized to monocyte numbers. Values are mean ±SEM, *n* = 15 per group. Significant changes are indicated where *p* < 0.05.

All biochemical variables are presented in Figure 1, Figure 2, Figure 3, Figure 4, Figure 5 and Figure 6. Significant findings were noted for several variables; there was a significant increase in serum Zonulin after four weeks of AL2 supplementation (*p* = 0.04), however, concentrations returned to baseline after eight weeks of supplementation (Figure 1). No significant change was observed for total white blood cell count for any of the groups (*p* > 0.05), but leukocyte composition was altered, with a significant increase in the lymphocytes fraction in the AA2 (*p* = 0.01 at week 4; *p* = 0.005 at week 8) and AL4 (*p* = 0.04 at week 4) groups (Figure 2B). Figure 2D shows a concomitant decrease in the monocyte fraction for AA2 (*p* = 0.03 at week 4; *p* = 0.02 at week 8) and AL4 (*p* = 0.03 at week 4). Monocytes were also decreased in absolute number (Figure 2E), with AA2 (*p* = 0.04 at week 4; *p* = 0.03 at week 8) and AL4 (*p* = 0.04 at week 8). Although not significant, there was a reduction in monocyte numbers with AA4 and AL2 not seen with the Placebo group. No difference was seen in blood granulocyte fraction (*p* > 0.05).

Whole blood was stimulated ex vivo with a TLR4 agonist to monitor the cytokine response. There were no significant changes with supplementation in the release of IL-6, IL-1β and IL-10 after a six-hour LPS exposure (Figure 3, Figure 4, Figure 5 and Figure 6), even after normalizing to monocyte numbers (note: monocytes are the most potent responders to LPS). Interestingly, there was an increase in systemic IL-6 with eight weeks of AA4 supplementation, as seen in the unstimulated samples (*p* = 0.04, Figure 5A). Systemic concentrations of the cytokine IL-10 was also slightly, but significantly increased with eight weeks of AL4 (*p* = 0.004) supplementation (Figure 6A). Only the AA2 group had an increase in TNF-α concentrations (on a per cell basis) with LPS stimulation (*p* = 0.04, Figure 3C). A small number of samples for TNF-α stimulated (*n* = 8), IL-10 unstimulated (*n* = 1), and IL-1β unstimulated (*n* = 5) were not available for data analysis due to values being too high or too low, relative to the standard curve. For IL-6, a significant number of unstimulated (*n* = 39) and stimulated (*n* = 92) samples were not available for analysis and this should be considered when interpreting the IL-6 data.

## 4. Discussion

The present study was designed to determine the overall impact of two different glyconutrient supplements on measures of gut health, immune function, and psychological well-being. The main findings of this study indicate that the glyconutrient supplement Ambrotose is (1) well-tolerated (only one adverse event of the 75 enrolled subjects and this event was noted to be mild in nature and involved gastrointestinal discomfort with use of the AL2 supplement); (2) results in no significant elevation in resting heart rate or blood pressure, as values remained similar across the eight-week study in all treatments; (3) is associated with higher overall well-being as compared to placebo, although values did not change significantly over time; (4) does not result in any change in SF-12 scores over an eight-week study period; (5) did not alter serum Zonulin concentrations or negatively affect hematological and immune parameters. Interestingly, supplementation resulted in a consistent reduction in blood monocyte numbers to a concentration that is still within the healthy range, and changes in specific cytokine responses, suggesting that, in a healthy population, the glyconutrient has the ability to affect components of the immune system.

Prior studies involving the glyconutrient Ambrotose have noted positive outcomes when assessing various surrogate markers of health. For example, Ambrotose glyconutrients have been associated with improvements in memory and subjective ratings of well-being in numerous studies [4,5,15,16]. With regards to immune modulation, marathon runners ingesting Advanced Ambrotose daily for 15 days prior to competition exhibited lower resting salivary chemokines, Gro-alpha and Gro-beta, compared to nonsupplemented counterparts; however, supplemented runners did not experience a reduction in these markers after exercise but did experience a reduction in the salivary chemokine, angiogenin, after exercise [17]. Alternatively, Ambrotose glyconutrients can also be offered with added antioxidants (Ambrotose AO). Studies utilizing Ambrotose AO demonstrated increased antioxidant capacity [2,6] and also suggest increased phagocytic activity of granulocytes [2].

In the present study, we did not note any significant improvements in subjective measures of well-being, as assessed through routine questionnaires. This is likely due to the fact that subjects in the present study were young and healthy, with most being engaged in regular exercise (average of 5–7 h per week, depending on treatment). Considering the starting values of these individuals for the various metrics, it is not surprising that significant improvements were not observed during the eight-week intervention period.

With regards to the biochemical measures, there were no dramatic effects observed, possibly due to the fact that the study participants were healthy and not experiencing “leaky gut” symptoms or immune disorders. We did observe a consistent decrease in monocyte number, with no change in total white blood cell count after supplementation. This result is interesting as it suggests that leukocyte composition might be altered by this glyconutrient [18]. Monocyte numbers are typically increased with chronic infection [19], but elevated concentrations are also associated with worsening of insulin sensitivity [20] and most recently noted to be a risk factor for cardiovascular disease [21]. Therefore, a lower overall monocyte percentage may be associated with improved health over time. It is worth mentioning here that monocyte numbers were higher in all Ambrotose groups as compared to Placebo at the baseline measurement (Figure 1), with a fairly dramatic decrease over the course of the eight-week intervention. While monocytes were reduced with treatment, this did not attenuate the body’s ability to respond to the LPS challenge, as evidenced by the cytokine response.

The most significant change seen in systemic cytokine concentrations, was an increase in IL-10, induced by AL4. IL-10 is a potent cytokine that plays a central role in limiting cellular adversity in order to support the body’s ability to maintain healthy tissues. This cytokine is also essential for intestinal health, as demonstrated by the induction of cellular adversity in the bowel of an IL-10-deficient mouse model [22], similar to what is seen in humans with polymorphism associated with IL-10 [23]. It also has neuro-health benefits and is implicated in various neurological situations [24,25]. The increase of IL-10 by a dietary component suggest that the glyconutrient might also benefit the gut microbiome (this was not tested in the current study), as gut-resident bacteria have been shown to exert beneficial effects through the induction of IL-10 [26] and it is therefore feasible that the prebiotic effect of Ambrotose is selecting for the enrichment of gut-beneficial microbes. The effect could be related to the acemannan content of the Ambrotose, as the microbiota has been shown to be favorably impacted by the aloe vera plant [27], with potential of acemannan as a prebiotic agent [28].

## 5. Conclusions

Our findings indicate that the glyconutrient supplement Ambrotose is very well-tolerated by healthy adults and may be associated with higher values on the Psychological General Well-Being Index as compared to a placebo. Also, Ambrotose supplementation can reduce circulating monocytes numbers and alter cytokine concentrations, suggesting that it effects immune function. These findings show Ambrotose supplementation is likely an immune system modulator as opposed to being an immune system stimulator. No clear differences were detected among the different treatment groups.

## Figures and Tables

**Table 1:** Characteristics of 75 healthy men and women.

Variable	Ambrotose Advanced 4 g (*n* = 15)	Ambrotose Advanced 2 g (*n* = 15)	Placebo (*n* = 15)	Ambrotose LIFE 4 g (*n* = 15)	Ambrotose LIFE 2 g (*n* = 15)	*p* Value
Age (years)	26.9 ± 5.4	26.3 ± 5.7	29.5 ± 11.6	30.2 ± 11.4	28.1 ± 7.4	0.70
Height (cm)	171.3 ± 10.3	174.0 ± 10.6	171.9 ± 8.2	170.8 ± 9.2	168.8 ± 9.6	0.68
Weight (kg)	73.7 ± 17.1	73.9 ± 17.3	78.4 ± 15.5	79.1 ± 15.5	69.7 ± 13.1	0.47
BMI (kg∙m^−2^)	24.9 ± 4.5	24.2 ± 4.1	26.4 ± 4.3	27.1 ± 4.8	24.4 ± 4.4	0.30
Waist Circumference (cm)	81.3 ± 10.4	80.2 ± 10.0	86.7 ± 12.6	87.2 ± 13.8	80.2 ± 8.4	0.21
Hip Circumference (cm)	101.8 ± 8.6	100.0 ± 8.8	103.2 ± 12.3	102.9 ± 10.4	100.0 ± 11.0	0.85
Waist/Hip Ratio	0.80 ± 0.06	0.80 ± 0.04	0.84 ± 0.10	0.85 ± 0.08	0.80 ± 0.04	0.12
Resting HR (bpm)	68.7 ± 12.8	64.5 ± 12.8	72.0 ± 10.2	71.5 ± 10.6	66.5 ± 13.4	0.39
Resting SBP (mm Hg)	120.5 ± 10.4	122.9 ± 12.0	125.6 ± 10.4	119.9 ± 13.0	117.8 ± 11.2	0.40
Resting DBP (mm Hg)	71.6 ± 8.7	73.8 ± 7.7	78.3 ± 8.3	76.1 ± 10.0	73.2 ± 10.0	0.29
Anaerobic Exercise (yrs)	4.4 ± 3.8	4.3 ± 5.2	6.1 ± 6.1	3.4 ± 4.2	4.2 ± 3.5	0.62
Anaerobic Exercise (hrs/wk)	2.8 ± 3.1	1.3 ± 1.2	2.1 ± 2.4	2.2 ± 2.5	1.8 ± 1.7	0.48
Aerobic Exercise (yrs)	7.6 ± 6.1	8.1 ± 7.1	8.7 ± 10.0	4.8 ± 4.5	7.5 ± 6.2	0.59
Aerobic Exercise (hrs/wk)	2.4 ± 1.4	3.0 ± 1.7	3.1 ± 2.0	2.6 ± 1.6	2.7 ± 1.3	0.69

Values are Mean ± SD. No differences of statistical significance noted for any variable (*p* > 0.05).

**Table 2:** Dietary data of 75 healthy men and women during the five days prior to each test day.

Variable	Ambrotose Advanced 4 g (*n* = 15)	Ambrotose Advanced 2 g (*n* = 15)	Placebo (*n* = 15)	Ambrotose LIFE 4 g (*n* = 15)	Ambrotose LIFE 2 g (*n* = 15)
Calories					
Week 0	2092.1 ± 864.1	2058.1 ± 477.8	1756.2 ± 245.2	2122.3 ± 798.6	2108.9 ± 599.8
Week 4	1836.5 ± 802.4	2092.3 ± 480.0	1687.4 ± 276.6	2281.8 ± 955.5	1994.0 ± 651.0
Week 8	2072.0 ± 932.6	2066.5 ± 480.3	1673.7 ± 326.9	2191.3 ± 868.0	2090.8 ± 673.3
Protein (g)					
Week 0	105.6 ± 77.1	88.0 ± 23.1	75.0 ± 26.2	96.6 ± 54.4	103.3 ± 33.2
Week 4	94.5 ± 75.2	90.5 ± 29.0	67.8 ± 18.1	99.1 ± 76.3	88.9 ± 30.5
Week 8	111.3 ± 79.7	98.6 ± 38.1	72.0 ± 21.7	108.3 ± 73.1	99.9 ± 37.7
Carbohydrate (g)					
Week 0	225.9 ± 74.1	240.8 ± 57.6	191.5 ± 50.4	209.1 ± 102.3	253.6 ± 80.1
Week 4	217.0 ± 84.5	245.7 ± 52.5	189.1 ± 40.2	223.8 ± 127.0	236.8 ± 83.3
Week 8	216.4 ± 95.7	228.7 ± 44.2	181.6 ± 49.8	205.2 ± 111.4	254.1 ± 80.8
Fiber (g)					
Week 0	21.4 ± 9.1	18.4 ± 4.5	15.0 ± 8.8	21.8 ± 12.3	23.9 ± 10.7
Week 4	19.3 ± 9.0	18.6 ± 5.9	14.0 ± 5.0	23.0 ± 16.0	22.9 ± 11.0
Week 8	19.3 ± 7.7	15.9 ± 4.0	14.7 ± 7.3	21.3 ± 15.3	22.3 ± 9.3
Sugar (g)					
Week 0	63.9 ± 23.8	87.5 ± 36.2	65.0 ± 30.8	77.4 ± 53.2	86.3 ± 30.2
Week 4	68.9 ± 34.3	84.2 ± 29.2	54.8 ± 22.8	80.2 ± 61.3	82.0 ± 39.5
Week 8	68.7 ± 48.1	87.9 ± 23.7	51.0 ± 25.1	68.2 ± 57.5	89.4 ± 34.8
Fat (g)					
Week 0	84.9 ± 37.5	82.8 ± 26.4	69.4 ± 14.5	84.6 ± 43.9	76.1 ± 29.3
Week 4	67.9 ± 37.4	83.3 ± 24.6	69.6 ± 17.5	86.7 ± 44.8	77.7 ± 28.9
Week 8	82.2 ± 41.8	84.8 ± 26.9	68.1 ± 18.1	86.2 ± 38.6	75.8 ± 26.3
Vitamin C (mg)					
Week 0	59.9 ± 40.6	61.5 ± 42.1	52.3 ± 47.2	56.2 ± 41.7	104.0 ± 86.5
Week 4	73.5 ± 43.3	54.3 ± 31.0	45.9 ± 29.0	68.0 ± 79.5	71.9 ± 56.1
Week 8	58.3 ± 31.3	60.7 ± 24.6	57.9 ± 46.9	65.0 ± 52.0	127.8 ± 122.8
Vitamin E (mg)					
Week 0	6.1 ± 6.7	3.7 ± 2.6	2.9 ± 2.8	6.7 ± 5.7	6.2 ± 3.4
Week 4	5.8 ± 6.2	3.6 ± 2.2	2.5 ± 1.2	5.3 ± 5.7	5.4 ± 4.7
Week 8	5.8 ± 5.3	4.6 ± 2.6	2.7 ± 1.7	10.6 ± 12.9	7.2 ± 4.5
Vitamin A (RE)					
Week 0	346.8 ± 212.9	368.4 ± 334.6	248.6 ± 307.8	437.4 ± 422.8	576.7 ± 618.7
Week 4	321.2 ± 210.8	386.8 ± 286.3	288.4 ± 252.2	578.3 ± 599.6	444.9 ± 302.5
Week 8	463.2 ± 345.2	413.3 ± 256.8	346.4 ± 236.7	778.6 ± 1187.8	540.0 ± 395.5

Values are Mean ± SD per day; No differences of statistical significance noted for any variable (*p* > 0.05). Note: the fiber content of the treatments was not considered in the diet analysis.

**Table 3:** Heart rate and blood pressure of 75 healthy men and women.

Variable	Ambrotose Advanced 4 g (*n* = 15)	Ambrotose Advanced 2 g (*n* = 15)	Placebo (*n* = 15)	Ambrotose LIFE 4 g (*n* = 15)	Ambrotose LIFE 2 g (*n* = 15)
Heart rate (bpm)					
Week 0	69.3 ± 13.3	65.7 ± 9.2	73.0 ± 13.9 *	69.8 ± 9.9	67.3 ± 14.3
Week 4	68.2 ± 11.4	66.8 ± 9.6	74.3 ± 13.3	70.9 ± 8.6	66.1 ± 15.9
Week 8	72.3 ± 14.8	73.0 ± 9.5	74.7 ± 11.6	77.7 ± 11.1	69.4 ± 15.4
Systolic Blood Pressure (mm Hg)					
Week 0	119.9 ± 15.2	119.7 ± 8.6	122.2 ± 10.8	118.6 ± 11.4	117.7 ± 15.4
Week 4	119.9 ± 13.7	122.7 ± 11.2	121.7 ± 14.2	123.1 ± 13.0	116.3 ± 15.0
Week 8	122.5 ± 11.5	118.4 ± 6.7	119.5 ± 9.2	122.3 ± 11.0	116.9 ± 14.2
Diastolic Blood Pressure (mm Hg)					
Week 0	72.1 ± 6.8	69.5 ± 8.9	78.1 ± 9.3 *	74.8 ± 9.9 *	71.3 ± 13.8
Week 4	72.7 ± 5.2	73.9 ± 8.7	78.5 ± 9.3	77.9 ± 10.3	72.3 ± 9.8
Week 8	72.7 ± 6.9	71.9 ± 5.9	77.7 ± 8.1	79.1 ± 11.8	74.5 ± 11.1
Values are Mean ± SD					

Values are Mean ± SD * Treatment effect for heart rate (HR) (*p* = 0.009), with values for placebo higher than for AA2 (treatment with 2 g of Advanced Ambrotose); Treatment effect for diastolic blood pressure (*p* = 0.002), with values for placebo and AL4 (treatment with 4 g of Ambrotose LIFE) higher than for AA2; No other effects of statistical significance were noted (*p* > 0.05).

**Table 4:** Subjective feelings of 75 healthy men and women.

Variable	Ambrotose Advanced 4 g (*n* = 15)	Ambrotose Advanced 2 g (*n* = 15)	Placebo (*n* = 15)	Ambrotose LIFE 4 g (*n* = 15)	Ambrotose LIFE 2 g (*n* = 15)
Attentive					
Week 0	69.7 ± 12.7	70.6 ± 16.5	76.5 ± 16.3	66.2 ± 17.7	76.1 ± 18.6
Week 4	67.3 ± 16.3	68.0 ± 10.2	68.3 ± 16.2	66.1 ± 17.1	67.4 ± 19.4
Week 8	66.1 ± 18.1	72.2 ± 14.3	72.2 ± 17.6	69.2 ± 18.0	71.3 ± 19.8
Tired					
Week 0	49.8 ± 26.7	35.4 ± 22.2	36.6 ± 26.3	47.1 ± 23.3	33.2 ± 22.2
Week 4	47.4 ± 27.4	42.5 ± 19.3	48.3 ± 31.4	43.7 ± 24.9	34.6 ± 25.3
Week 8	43.6 ± 29.0	30.0 ± 20.9	43.4 ± 27.0	34.8 ± 20.9	30.1 ± 21.4
Alert					
Week 0	70.3 ± 17.2	67.6 ± 18.5	74.7 ± 16.6	68.1 ± 17.5	72.4 ± 19.8
Week 4	58.4 ± 17.6	64.9 ± 10.4	67.5 ± 16.5	65.9 ± 16.9	65.7 ± 21.8
Week 8	64.6 ± 14.6	68.1 ± 17.1	69.2 ± 18.3	68.3 ± 18.5	71.7 ± 17.6
Groggy					
Week 0	32.7 ± 29.9	22.4 ± 24.3	29.4 ± 27.2	36.5 ± 27.6	21.9 ± 21.9
Week 4	40.9 ± 26.6	32.4 ± 23.8	34.3 ± 26.0	27.5 ± 24.1	23.7 ± 24.2
Week 8	32.4 ± 28.4	29.4 ± 23.6	27.3 ± 15.3	16.9 ± 16.2	23.9 ± 20.1
Focused					
Week 0	66.2 ± 15.1	63.3 ± 18.1	69.7 ± 19.0	63.5 ± 19.7	72.7 ± 18.9
Week 4	63.9 ± 16.9	72.7 ± 10.3	71.7 ± 15.3	68.3 ± 19.6	65.4 ± 19.2
Week 8	64.4 ± 19.5	69.7 ± 16.1	68.0 ± 18.1	69.6 ± 17.8	73.9 ± 16.6
Sluggish					
Week 0	31.2 ± 29.2	22.1 ± 25.9	24.1 ± 21.7	36.7 ± 27.5	21.3 ± 20.1
Week 4	39.1 ± 27.9	29.4 ± 23.3	29.1 ± 22.2	25.0 ± 23.9	25.1 ± 22.5
Week 8	33.6 ± 29.5	27.0 ± 24.9	28.2 ± 22.0	20.3 ± 17.2	27.5 ± 23.1
Energetic					
Week 0	43.9 ± 21.2	56.7 ± 15.9	59.8 ± 21.6	53.6 ± 19.6	66.7 ± 22.4
Week 4	53.6 ± 19.0	60.1 ± 19.5	51.1 ± 29.1	60.8 ± 19.3	67.1 ± 18.5
Week 8	59.9 ± 15.8	65.3 ± 18.4	62.7 ± 25.2	65.8 ± 16.9	65.3 ± 17.7
Lethargic					
Week 0	36.2 ± 27.3	14.2 ± 20.9	20.2 ± 16.1	31.4 ± 24.5	21.9 ± 20.6
Week 4	35.6 ± 23.0	17.3 ± 18.5	27.7 ± 24.0	17.3 ± 15.0	24.9 ± 25.6
Week 8	27.4 ± 23.7	26.0 ± 31.0	19.6 ± 17.9	12.7 ± 12.0	24.1 ± 20.8
Enthusiastic					
Week 0	53.8 ± 24.4	56.7 ± 17.7	49.8 ± 24.9	58.9 ± 24.4	70.3 ± 20.9
Week 4	59.6 ± 17.5	53.1 ± 22.9	52.8 ± 27.6	61.5 ± 25.2	66.7 ± 22.0
Week 8	61.4 ± 17.8	69.1 ± 11.7	62.7 ± 22.4	68.8 ± 20.6	66.8 ± 20.0
Sore					
Week 0	25.6 ± 29.9	27.9 ± 23.6	20.8 ± 26.3	24.2 ± 24.2	19.3 ± 22.0
Week 4	34.4 ± 30.3	30.7 ± 29.9	21.3 ± 24.2	21.8 ± 26.1	21.7 ± 25.3
Week 8	29.0 ± 29.3	29.7 ± 29.8	14.9 ± 21.8	22.1 ± 20.0	17.9 ± 20.7
Well-Rested					
Week 0	49.4 ± 24.1	54.3 ± 21.5	50.5 ± 27.7	56.1 ± 27.9	55.7 ± 26.0
Week 4	45.0 ± 19.9	58.3 ± 20.4	44.6 ± 27.2	58.7 ± 30.3	56.6 ± 26.5
Week 8	56.3 ± 27.2	62.5 ± 21.5	58.1 ± 25.8	63.9 ± 22.7	66.9 ± 24.4
Fatigued					
Week 0	30.9 ± 29.9	29.3 ± 20.5	33.4 ± 25.4	24.7 ± 24.5	26.1 ± 25.1
Week 4	33.4 ± 24.7	22.5 ± 21.7	30.4 ± 27.4	24.3 ± 20.7	31.1 ± 24.0
Week 8	33.7 ± 28.9	28.7 ± 20.5	34.0 ± 26.7	15.3 ± 17.8	24.3 ± 19.8
Sickly					
Week 0	17.4 ± 22.4	17.1 ± 24.8	5.1 ± 9.8	7.7 ± 11.5	17.4 ± 28.8
Week 4	11.6 ± 14.8	12.6 ± 14.5	15.5 ± 21.5	5.1 ± 5.3	12.1 ± 22.5
Week 8	16.9 ± 22.7	19.5 ± 25.1	14.8 ± 24.7	15.8 ± 19.3	22.1 ± 28.8
Memory and Cognition					
Week 0	61.4 ± 23.3	71.8 ± 15.4	64.1 ± 30.3	67.7 ± 21.5	71.4 ± 21.5
Week 4	66.1 ± 16.7	62.9 ± 21.0	67.2 ± 27.9	69.1 ± 20.6	65.0 ± 19.5
Week 8	64.6 ± 17.4	64.3 ± 23.7	71.2 ± 15.2	74.1 ± 14.7	71.1 ± 15.2
Mental Stress					
Week 0	39.1 ± 28.2	33.4 ± 28.5	30.9 ± 30.6	36.3 ± 21.4	29.8 ± 25.3
Week 4	33.4 ± 26.2	27.5 ± 22.0	53.7 ± 26.3	35.9 ± 24.5	42.7 ± 26.3
Week 8	43.5 ± 28.0	30.7 ± 25.5	45.3 ± 29.2	35.3 ± 26.1	42.5 ± 27.3

Values are Mean ± SD; No treatment, time, or treatment × time effects were noted (*p* > 0.05).
